# The interplay between bile acid metabolism and gut microbiome in biliary tract cancers

**DOI:** 10.3389/frmbi.2026.1774429

**Published:** 2026-05-28

**Authors:** Ifeoma Ike, Farzad Teymouri, Christiana Crook, Sofia Guzman, Max Hazeltine, Dani Castillo, Daneng Li, Gagandeep Brar

**Affiliations:** 1 Department of Medicine, Eisenhower Health, Rancho Mirage, CA, United States; 2 Department of Medical Oncology & Therapeutics Research, City of Hope Comprehensive Cancer Center, Duarte, CA, United States

**Keywords:** bile acid, biliary tract cancer, extrahepatic cholangiocarcinoma, gallbladder carcinoma, intrahepatic cholangiocarcinoma, microbiome

## Abstract

The gut microbiota and bile acids (BAs) exist in a tightly regulated, bidirectional relationship that influences host metabolism, immune function, and disease. Primary BAs synthesized in the liver are chemically transformed by intestinal microbes into a diverse pool of secondary BAs, which exert antimicrobial effects and activate host signaling pathways including Farnesoid X Receptor (FXR), Takeda G protein–coupled receptor 5 (TGR5), and sphingosine-1-phosphate receptor 2 (S1PR2). These pathways regulate BA homeostasis, epithelial barrier integrity, inflammation, and carcinogenesis. Disruption of this BA–microbiome axis has been implicated in biliary tract cancers (BTCs), a group of aggressive malignancies with rising global incidence and limited therapeutic options. Secondary BAs and BA receptor signaling contribute to tumor initiation and progression through NF-κB activation, oxidative stress, and altered cell survival, whereas reduced FXR signaling and obstructed enterohepatic circulation further promote inflammatory dysregulation. Emerging evidence demonstrates that microbial dysbiosis and altered BA metabolism are associated with distinct BTC microbial profiles, enriched in taxa such as Fusobacterium, Salmonella, Prevotella, and Actinomyces, alongside depletion of commensals including Lactobacillus. These taxa influence inflammatory signaling, BA transformation, and epithelial injury, contributing to carcinogenesis. Microbiome–BA interactions also shape anti-tumor immunity and responses to immune checkpoint inhibitors (ICIs). Specific microbial signatures—particularly enrichment of Lachnospiraceae, Erysipelotrichaceae, Bacteroidetes, and Alistipes—correlate with enhanced immune activation and improved clinical outcomes in hepatobiliary cancers. Modulation of gut microbiota through antibiotics, probiotics, or fecal microbiota transplantation can influence BA composition, immune surveillance, and therapeutic efficacy. Collectively, these data highlight the central role of the BA–microbiome axis in BTC pathogenesis and treatment response. Microbial and BA metabolite profiling represent promising avenues for biomarker development, while targeted manipulation of BA signaling and microbial ecology offers potential therapeutic strategies to improve BTC outcomes.

## Introduction

The gut microbiota and bile acids engage in a tightly regulated, bidirectional relationship that plays a fundamental role in host metabolism, immunity, and disease. Gut microbiota refers to a collection of microorganisms in the gastrointestinal tract including bacteria, archaea, viruses, and eukaryotes. BAs are synthesized in the liver from cholesterol and further modified by intestinal microbes into a variety of secondary forms with distinct biological properties. These microbial transformations substantially expand the chemical diversity of the BA pool and influence host physiology by modulating antimicrobial defenses, metabolic homeostasis, epithelial barrier function, and receptor-mediated signaling pathways that govern inflammation, immunity, and carcinogenesis (Guo et al., 2022). In turn, BAs function as signaling molecules, antimicrobial agents, and metabolic regulators, shaping the composition of the microbiome and modulating host gene expression, thus creating the BA–microbiome axis (Kim et al., 2007; Fiorucci et al., 2009; Vavassori et al., 2009; Guo et al., 2022).

**Figure 1:**
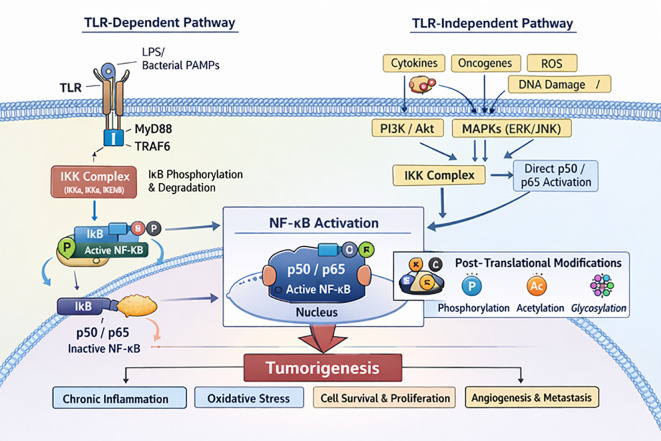
Canonical NF-κB signaling pathways in tumorigenesis: TLR-dependent and TLR-independent activation mechanisms. This schematic illustrates NF-κB activation through TLR-dependent signaling (LPS/PAMP–TLR–MyD88/TRAF6–IKK complex–IκB degradation) and TLR-independent mechanisms involving cytokines, oncogenes, ROS, DNA damage, and PI3K/Akt or MAPK pathways converging on IKK or directly on p50/p65. Both routes culminate in nuclear translocation of the canonical NF-κB heterodimer (p50/p65), whose transcriptional activity is fine-tuned by post-translational modifications including phosphorylation, acetylation, and glycosylation. Activated NF-κB drives gene programs linked to chronic inflammation, oxidative stress responses, cell survival, proliferation, angiogenesis, and metastasis, thereby promoting tumorigenesis. Abbreviations: IKK, IκB kinase; LPS, lipopolysaccharide; NF-κB, nuclear factor-kappa B; PAMPs, pathogen-associated molecular patterns; ROS, reactive oxygen species; TLR, toll-like receptor.

Disruption of this BA–microbiome axis has been implicated in several diseases such as cholecystitis, primary sclerosing cholangitis, and BTCs (Ridlon et al., 2016; Sato et al., 2021; Ye et al., 2023). In the setting of liver and biliary disease, systemic endotoxemia has been documented in patients with obstructive jaundice or cirrhosis—conditions that often complicate biliary tract tumors—with endotoxin detectable in the circulation in up to 70% of affected individuals, likely due to increased intestinal permeability and impaired hepatic clearance (van Santvoort et al., 2008). Although direct prevalence studies of systemic endotoxemia or bacterial DNA translocation in biliary tract cancer cohorts are limited, these patterns of microbial translocation observed in related hepatic and gastrointestinal cancers highlight a plausible mechanism by which gut barrier dysfunction and endotoxin exposure could contribute to systemic inflammation, immune dysregulation, and cancer progression. BTCs, which encompass intrahepatic cholangiocarcinoma, extrahepatic cholangiocarcinoma, and gallbladder carcinoma, are aggressive malignancies with rising incidence and limited therapeutic options (Roa et al., 2022; Ye et al., 2023). Emerging data suggest that alterations in microbial composition and BA metabolism may influence BTC pathogenesis, progression, and response to immunotherapy (**Figure 1**) (Sharma et al., 2007; Tsuchiya et al., 2018). This review examines the bidirectional BA–gut microbiome relationship and its implications in BTCs.

## Bile acid overview

BAs are the final products of cholesterol metabolism and represent a principal route for cholesterol elimination in humans. Their synthesis not only facilitates bile formation but also enables the excretion of phospholipids, cholesterol, xenobiotics, and endogenous waste products (Ridlon et al., 2016; Guo et al., 2022).

BAs are synthesized in the liver from modification of the sterol ring of cholesterol followed by side chain cleavage reactions to form primary BAs, mainly cholic acid and chenodeoxycholic acid. BAs are then conjugated with glycine or taurine to form bile salts (Rembacz et al., 2010; Ridlon et al., 2016). These bile salts aggregate with phospholipids and cholesterol to form mixed micelles, which are stored in the gallbladder and later released into the small intestine, where they aid in the emulsification and absorption of dietary fats and fat-soluble nutrients. Following their intestinal function, the majority of BAs are actively reabsorbed in the ileum and return to the liver through the portal vein (Ridlon et al., 2016). This highly efficient enterohepatic circulation plays a central role not only in digestion and detoxification but also in preserving metabolic balance (Chiang, 2013; Sannasiddappa et al., 2017).

In the intestine, bile salt hydrolases deconjugate primary BAs, increasing their hydrophobicity and epithelial permeability (Ridlon et al., 2016). Accumulation of these unconjugated bile acids within the biliary tree promotes cholangiocyte membrane injury, mitochondrial dysfunction, and activation of inflammatory pathways including NF-κB. Unconjugated BAs such as cholic acid and chenodeoxycholic acid, are then converted into the secondary BAs deoxycholic acid and lithocholic acid via enzymes encoded by the bile acid–inducible operon (bai) (Ridlon et al., 2016; Song et al., 2018; Guo et al., 2022). These secondary BAs are more cytotoxic and genotoxic, inducing oxidative DNA damage and activating proliferative signaling pathways such as TGR5, thereby enhancing cholangiocyte proliferation and resistance to apoptosis. Additional oxidation and epimerization reactions mediated by microbial hydroxysteroid dehydrogenases modify BA receptor activity, including FXR signaling, altering inflammatory tone and BA homeostasis. Collectively, these microbial transformations shift the BA pool toward more hydrophobic and pro-inflammatory species, fostering epithelial injury, chronic inflammation, and proliferative signaling that support biliary tract carcinogenesis (Ridlon et al., 2016).

## The bile acid–microbiome axis

Microbial communities shape the BA pool. Differentiation and/or certain levels of BAs induce an overgrowth of bacteria and potential pathogens (di Gregorio et al., 2021). Secondary BAs exhibit potent detergent-like effects on bacterial membranes and can act as bacteriostatic or bactericidal agents in a concentration-dependent manner. Accumulation of primary BAs can lower the intracellular pH in certain bacterial species, leading to a major disturbance in transmembrane electrical potential and subsequent inhibition in bacterial growth (di Gregorio et al., 2021; Wu et al., 2025).

In addition to antimicrobial roles, BAs serve as ligands for host receptors such as FXR and TGR5 (Wu et al., 2025). An intact gut epithelial barrier limits systemic exposure to luminal microbial products while fostering immune tolerance through induction of regulatory T cells and production of anti-inflammatory cytokines such as IL-10 and TGF-β, processes partly mediated by short-chain fatty acids and bile acid–activated receptors including FXR and TGR5 (Rooks and Garrett, 2016). Maintenance of this barrier is critical for restraining mucosal and hepatobiliary inflammation, whereas its disruption permits microbial translocation and endotoxin-driven cytokine signaling that may contribute to chronic inflammation and tumor-promoting conditions within the biliary tract.

FXR activation regulates BA synthesis and transport, and maintains intestinal epithelial barrier function (Erice et al., 2018; Cossiga et al., 2023). By suppressing cholesterol 7 alpha-hydroxylase —the key enzyme governing BA synthesis—FXR exerts negative feedback control on BA production. Additionally, its activation enhances the expression of transporters that facilitate BA efflux, thereby mitigating hepatocellular toxicity (Wu et al., 2025; Yan et al., 2025). TGR5, expressed in non-parenchymal cells including cholangiocytes, modulates inflammation, metabolism, and cell proliferation in a context-dependent manner (Fiorucci et al., 2009; Wu et al., 2025). Importantly, conjugated and unconjugated BAs can differentially activate these receptors, with unconjugated BAs more strongly activating FXR and conjugated forms often favoring TGR5 or pro-inflammatory signaling via Sphingosine-1-phosphate receptor 2 (S1PR2) (Fiorucci et al., 2009; Song et al., 2018; Wu et al., 2025). S1PR2 has been implicated in promoting inflammatory and fibrotic responses, especially within cholangiocytes and hepatic stellate cells (Wu et al., 2025). Its signaling activity may play a contributory role in liver damage and the development of cholangiocarcinoma (Thibaut and Bindels, 2022).

## Bile acids in tumor biology

Beyond their roles in digestion and microbial regulation, BAs influence tumor biology through their actions on host signaling pathways. Among the most studied are signaling pathways involving FXR and TGR5, both of which are directly activated by BAs, particularly secondary BAs, and implicated in cancer pathogenesis (Cossiga et al., 2023; Wu et al., 2025).

FXR acts as a tumor suppressor in hepatocytes and cholangiocytes (Kim et al., 2007). Its activation by unconjugated BAs, mainly chenodeoxycholic acid and cholic acid downregulates BA synthesis, reduces lipogenesis, and induces an anti-inflammatory process for gene expression (Kim et al., 2007). In hepatocytes, FXR activation suppresses BA synthesis via repression of CYP7A1 (Kim et al., 2007). In cholangiocarcinoma, FXR expression is markedly reduced in tumor tissue compared to adjacent healthy liver tissue, with lower FXR expression correlating with poorer differentiation, advanced tumor staging, and increased vascular invasion (Bernstein et al., 2009; Dai et al., 2013). Pharmacologic activation of FXR, particularly with obeticholic acid, has been shown to inhibit proliferation and migration of cholangiocarcinoma cell lines and suppress tumor growth in xenograft murine models (Dai et al., 2013). These BTC-specific findings support a context-dependent tumor-suppressive role of FXR in cholangiocarcinoma. However, FXR signaling can differ between hepatocytes and cholangiocytes and across ligand classes, and tumor-suppressive effects should not be generalized to all BAs.

TGR5, which is activated by conjugated BAs, appears to have a context-specific role (Song et al., 2018; Jia et al., 2020). In non-malignant cholangiocytes, TGR5 activation increases intracellular cAMP and promotes cell survival and protects against BA-induced injury through enhancement of the “bicarbonate umbrella”, a chloride/bicarbonate exchange which maintains an alkaline microenvironment at the apical cell surface and protects cholangiocytes from BA toxicity (Beuers et al., 2010). TGR5 is preferentially activated by secondary BAs, particularly lithocholic acid and deoxycholic acid, with activity also observed for conjugated derivatives (Fiorucci et al., 2009). However, in malignant cells, TGR5 activation contributes to proliferation, resistance to apoptosis, and increased production of reactive oxygen species, creating a pro-tumorigenic environment.

Conjugated BAs such as taurocholic acid have also been shown to activate S1PR2, further amplifying pro-inflammatory and oncogenic pathways through COX-2 upregulation and increased prostaglandin E2 production (Wang et al., 2017; Song et al., 2018). In murine cholestasis models (supportive, non-BTC), S1PR2 activation promoted cholangiocyte proliferation and liver injury via ERK1/2 and AKT signaling and increased COX-2 expression. Although direct BTC models are limited, these pathways overlap with oncogenic signaling networks relevant to cholangiocarcinoma, suggesting potential extrapolative relevance to tumor-associated ductular proliferation.

Another signaling axis involved in BAs impact on tumor biology is nuclear factor-kappa B (NF-κB), a master regulator of inflammation and cell survival (Wang et al., 2017). *In vitro* studies demonstrate that unconjugated BAs inhibit NF-κB activation and suppress cholangiocarcinoma cell proliferation at higher concentrations, whereas conjugated BAs activate NF-κB, enhance IL-6 and COX-2 expression, and promote tumor growth (Wang et al., 2017). **Figure 1** depicts the signaling pathways involved in more detail (Zhang et al., 2021).

If any of these pathways are dysregulated or in an inflammatory state, recruitment of immune-inflammatory cells and abnormal production of cytokines and acute-phase reactants will occur (Zhao et al., 2025). In scenarios such as cancer, a tumor can obstruct bile flow thereby slowing the enterohepatic circulation of BAs and other biliary metabolites (Staley et al., 2017; Pezzino et al., 2022).

## The interplay between bile acids, microbiome, and biliary tract cancers

BTCs have been increasingly associated with microbiota dysbiosis (Sharma et al., 2007; Ye et al., 2023). Alterations in microbial communities and their metabolic products, particularly BAs, are now recognized as potential contributors to BTC pathogenesis (Song et al., 2018; Jia et al., 2020). Chronic microbial dysbiosis and persistent infections within the biliary system and gastrointestinal tract contribute to carcinogenesis through several mechanisms, including sustained inflammation, microbial production of carcinogenic metabolites, and alterations in host epigenetic regulation. Molecular analyses have revealed distinct microbial profiles in the bile of patients with biliary tract malignancies compared to those with non-malignant conditions (Sharma et al., 2007). Prevalent bacteria in these microbial profiles include *Actinomyces, S. typhi, Fusobacterium, Campylobacter, Helicobacter pylori*, and *Prevotella* (Endt et al., 2010; Kamada et al., 2013; Saab et al., 2021; Ye et al., 2023).

Several studies have correlated higher levels of pathogenic bacteria to BTC. In a paper by Avilés-Jiménez et al. where 100 patients with extrahepatic cholangiocarcinoma had their DNA extracted for microbiota characterization, *Fusobacterium, Prevotella*, *Helicobacter pylori* and *Campylobacter* were found to be highly present, suggesting their involvement in BTC through an unregulated inflammatory response involving a reduction in short-chain fatty acid producers and potentially impairing epithelial barrier integrity (Tan et al., 2014; Avilés-Jiménez et al., 2016; Saab et al., 2021). *Proteobacteria* (which includes *Campylobacter*) was found to account for approximately 60% of the microbial population within the bile duct—a composition closely resembling that of the small intestinal microbiota—alluding to a potential relationship between intestinal microbiota and the pathogenic environment in the biliary tract system (White et al., 2006; Kamada et al., 2013; Wu et al., 2013). While direct BA measurements were not performed in this study, Proteobacteria expansion is broadly associated (shared across hepatobiliary malignancies) with enhanced BA deconjugation capacity and inflammatory signaling (Avilés-Jiménez et al., 2016; Jia et al., 2020). These findings, however, are specific to extrahepatic disease and should not be extrapolated to intrahepatic cholangiocarcinoma without caution.

In contrast, intrahepatic cholangiocarcinoma has been more strongly linked to gut microbial and BA metabolic alterations rather than localized biliary colonization. Jia et al. demonstrated distinct gut microbiota profiles between patients with intrahepatic cholangiocarcinoma, hepatocellular carcinoma, and healthy individuals through 16S rRNA sequencing. Among 84 patients, 24 patients with intrahepatic cholangiocarcinoma had enrichment of *Lactobacillus*, *Actinomyces*, and *Peptostreptococcaceae*, with the first two genera showing potential diagnostic value in distinguishing intrahepatic cholangiocarcinoma from hepatocellular carcinoma or cirrhosis. Compared to patients with hepatocellular carcinoma or healthy individuals, patients with intrahepatic cholangiocarcinoma had an increase in tauroursodeoxycholic acid, a conjugated BA, which positively correlated with increased levels of *Lactobacillus* and *Actinomyces* (Jia et al., 2020). This finding supports a BA-gut microbiota axis in iCCA, though it derives from a mixed hepatobiliary cohort and thus cannot be considered exclusive to iCCA.

Other studies have reported an increased risk of gallbladder cancer in patients with cholelithiasis who also carry chronic *S. typhi* (Tsuchiya et al., 2018). This is supported by higher positivity for Vi antibodies (IgG) that target the Vi polysaccharide antigen in *S. typhi*. BA profiles in patients with gallbladder cancer also show marked shifts, with elevated levels of secondary BAs such as DCA and LCA and reduced levels of primary BAs (Sharma et al., 2007). Kim et al. showed that *S. typhi* infection binds to sulfated glycans on liver sinusoidal endothelial cells and gallbladder epithelial cells leading to a reduction in BAs, thus promoting *S. typhi* pathogenicity (Tsuchiya et al., 2018; Lee et al., 2020; Kim et al., 2025). Elevated secondary BAs are a shared pro-carcinogenic feature observed across gastrointestinal cancers, where they promote oxidative stress, NF-κB activation, and epithelial proliferation; however, in GBC, these alterations occur in the context of gallstones and chronic bacterial biofilm formation, suggesting a distinct gallbladder-specific inflammatory niche (Ridlon et al., 2016). **Table 1** highlights specific strains of bacteria that have been associated with BTCs.

**Table 1:** Summary of mentioned bacteria/taxa and their relevance in BTCs.

Bacteria/Taxa	Role/relevance to BTC	References
*Fusobacterium*	Enriched in BTC; induce unregulated inflammatory response and promote carcinogenesis	Saab et al, Kamada et al., Endt et al (Endt et al., 2010; Kamada et al., 2013; Saab et al., 2021)
*Prevotella*	Enriched in BTC; linked to pro-inflammatory activity	Saab et al, Kamada et al., Guo et al (Kamada et al., 2013; Saab et al., 2021; Guo et al., 2022)
*Campylobacter*	Enriched in BTC; contribute to tumorigenesis via inflammation	Avilés-Jiménez et al, Wu et al (Wu et al., 2013; Avilés-Jiménez et al., 2016)
*Lachnospiraceae bacterium-GAM79*	SCFA producer (butyrate); enhances immune response; participates in primary to secondary BA conversion; potential probiotic/FMT candidate	Routy et al., Mao et al (Routy et al., 2018; Mao et al., 2021)
*Erysipelotrichaceae bacterium-GAM147*	SCFA producer; supports Treg differentiation and anti-inflammatory cytokine production; may improve immunotherapy efficacy	Routy et al., Mao et al (Routy et al., 2018; Mao et al., 2021)
*Bacteroidetes*	Higher Bacteroidetes/Firmicutes ratio associated with better response to immune checkpoint inhibitors; impacts gut microbial balance	Zhu et al., Jin et al., Guo et al (Jin et al., 2019; Guo et al., 2022; Zhu et al., 2024)
*Alistipes (general)*	Produces SCFAs (propionate, acetate); modulates immune responses; enriched in durable clinical benefit group; associated with better overall survival/progression free survival; may influence BA metabolism	Zhu et al., Jin et al (Jin et al., 2019; Zhu et al., 2024)
*Alistipes putredinis*	Enriched in patients with favorable immunotherapy response; SCFA producer	Zhu et al., Jin et al (Jin et al., 2019; Zhu et al., 2024)
*Alistipes dispar*	Enriched in durable clinical benefit group; support anti-inflammatory and immunomodulatory effects	Zhu et al., Jin et al (Jin et al., 2019; Zhu et al., 2024)
*Alistipes onderdonkii*	Correlated with clinical benefit in other cancers; potential predictor of immunotherapy response in BTC	Zhu et al., Jin et al (Jin et al., 2019; Zhu et al., 2024)
*S. typhi*	Correlated with chronic biliary colonization, particularly in gallstone disease; persistent infection may promote inflammation-driven dysplasia and increase cholangiocarcinoma risk	Endt et al., Kim et al, Lee et al (Endt et al., 2010; Lee et al., 2020; Kim et al., 2025)
*Lactobacillus*	Typically considered a protective commensal genus; may reflect loss of barrier-supportive, anti-inflammatory microbial communities	Guo et al., Ridlon et al., Degirolamo et al., Yan et al (Degirolamo et al., 2014; Ridlon et al., 2016; Guo et al., 2022; Yan et al., 2025)
*Bifidobacterium*	Typically considered a protective commensal genus; may reflect loss of barrier-supportive, anti-inflammatory microbial communities	Degirolamo et al., Yan et al (Degirolamo et al., 2014; Yan et al., 2025)
*Actinomyces*	Occasionally isolated in biliary infections; may contribute to chronic biliary inflammation, creating a microenvironment that favors malignant transformation	Jia et al., Saab et al., Kamada et al., Endt et al (Endt et al., 2010; Kamada et al., 2013; Jia et al., 2020; Saab et al., 2021)
*Peptostreptococcaceae*	Enrichment reported in certain gastrointestinal malignancies; may influence BA metabolism and inflammatory signaling pathways relevant to BTC progression	Jia et al., Ye et al (Jia et al., 2020; Ye et al., 2023)
*Helicobacter pylori*	Detected in bile and biliary epithelium in some studies; suspected to promote chronic inflammation and epithelial injury contributing to BTC pathogenesis	Guo et al., Avilés-Jiménez et al., Ye et al (Avilés-Jiménez et al., 2016; Guo et al., 2022; Ye et al., 2023)

BTC, biliary tract cancer; FMT, fecal microbiota transplant; SCFA, short-chain fatty acid; T-reg, T-regulatory.

## Role in immune modulation and resistance mechanisms

Recent studies have uncovered key links between the gut microbiota, BA metabolism, and immune regulation in liver and biliary malignancies. A notable discovery involves the interaction between primary BAs and hepatic natural killer T (NKT) cells through the CXCL16–CXCR6 axis (Ma et al., 2018; Wu et al., 2025). In murine models, primary BAs promote expression of CXCL16 on liver sinusoidal endothelial cells, leading to recruitment and activation of CXCR6^+^ NKT cells, which exert antitumor effects (Wu et al., 2013). This immune surveillance mechanism is suppressed by secondary BAs such as DCA and LCA (Wu et al., 2013).

Manipulating the microbiota to favor the accumulation of primary BAs, such as by using antibiotics to deplete Gram-positive BA-transforming bacteria, can enhance NKT cell–mediated tumor suppression. These findings have been validated in human cohorts, including the TIGER-LC study, where high levels of chenodeoxycholic acid (a primary BA) correlated with higher CXCL16 expression and lower glycolithocholate levels (Chaisaingmongkol et al., 2017; Ma et al., 2018; Wu et al., 2025).

A review on nuclear receptor signaling and its role in BA-microbiome axis by *Yan et al*, showed that reduction of certain microbiota, such as Lactobacillus and Bifidobacterium can lead to FXR depletion and thereby suppress primary BA synthesis, specifically cholic acid/chenodeoxycholic acid synthesis, promoting inflammatory dysregulation resulting in hepatobiliary and intestinal diseases (Yan et al., 2025).

Although much of the mechanistic work derives from preclinical and non-biliary tumor models, the convergence of lipid signaling, microbial metabolism, and immune regulation suggests that fatty acid amide hydrolase and related endocannabinoid system components represent modulators of tumor behavior and treatment response within inflammation-driven cancers. Fatty acid amide hydrolase is a membrane-associated serine hydrolase that terminates signaling of endogenous amidated lipids, including the endocannabinoid anandamide and the related N-acylethanolamines palmitoylethanolamide and oleoylethanolamide, thereby regulating tone within the endocannabinoid system (Cravatt et al., 1996 et al., 1996; V et al., 2004). Through modulation of cannabinoid receptor 1, cannabinoid receptor 2, and peroxisome proliferator–activated receptor-α signaling, these lipid mediators influence metabolic homeostasis, neuroinflammation, nociception, and cellular proliferation (D, 2003). In cancer biology, altered fatty acid amide hydrolase expression or activity has been associated with dysregulated amidated lipid levels, which can variably affect tumor growth, apoptosis, angiogenesis, and immune cell recruitment in a context-dependent manner; in several malignancies, reduced fatty acid amide hydrolase activity enhances endocannabinoid signaling and may suppress proliferation via cannabinoid receptor 1/cannabinoid receptor 2-mediated pathways, whereas in others, endocannabinoid system activation has been linked to tumor progression and immune evasion (Maccarrone et al., 2015; Morena et al., 2016). Emerging evidence further highlights bidirectional crosstalk between the commensal gut microbiome and the endocannabinoid system. Microbial metabolites—including short-chain fatty acids and diet-derived polyunsaturated fatty acids —can modulate endocannabinoid tone by influencing N-acylethanolamine synthesis and degradation, intestinal permeability, and systemic inflammatory signaling (M et al., 2010; S and V, 2013; Cani et al., 2014). Conversely, host endocannabinoid system activity shapes gut barrier integrity and microbial composition, forming a feedback loop that integrates metabolic and immune cues. In oncologic settings, this microbiome–endocannabinoid axis may influence cancer progression and therapeutic responsiveness by altering inflammatory cytokine networks, myeloid cell polarization, oxidative stress, and resistance to chemotherapy or immunotherapy (Sido et al., 2016).

## Interaction between immunotherapy, microbiome, and BTC

**Figure 2:**
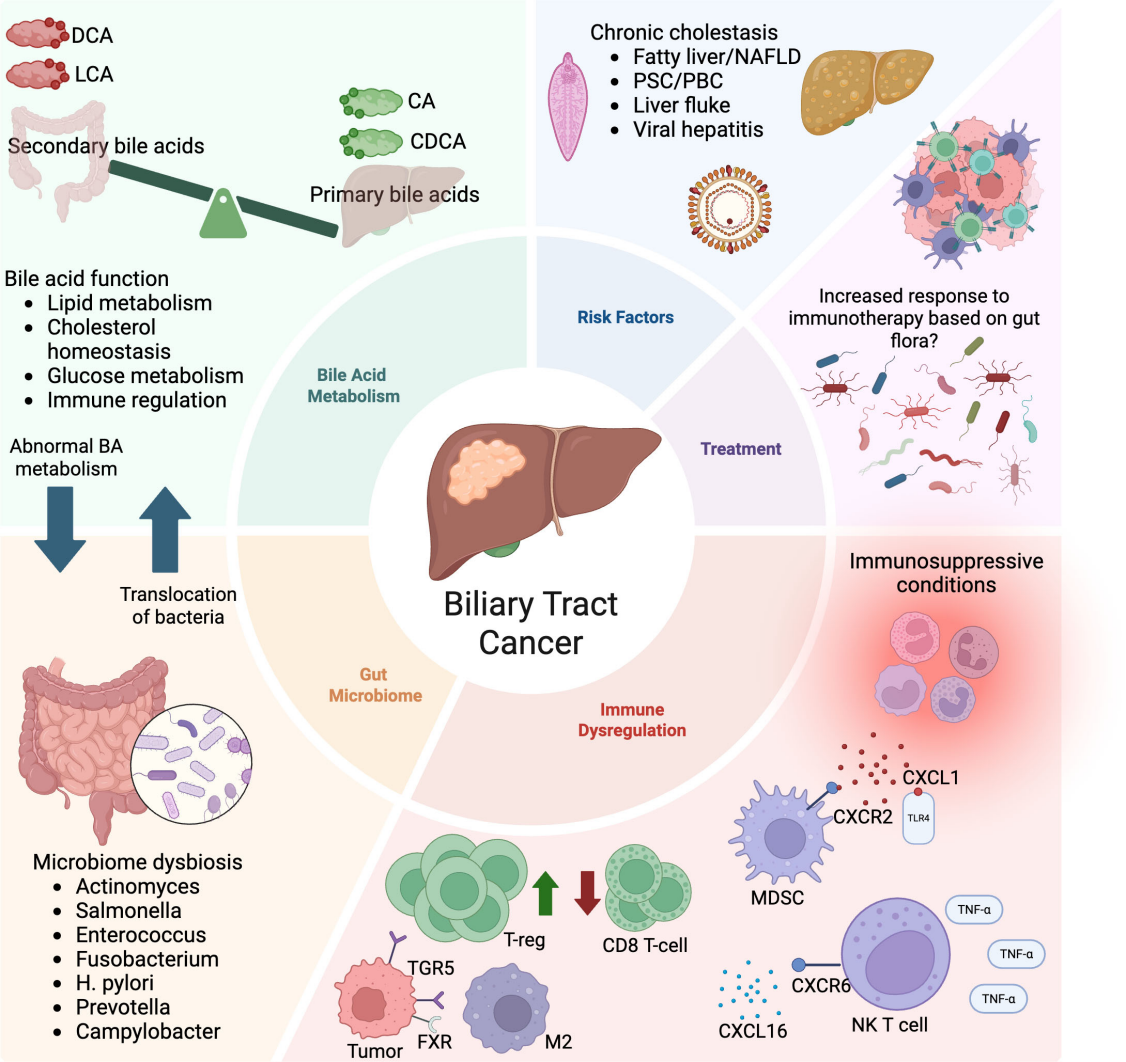
Overview of the interplay between BA metabolism, microbiome and immune dysregulation in BTC. Abbreviations: BA, bile acid; BTC, biliary tract cancer; CDCA, chenodeoxycholic acid; CA, cholic acid; DCA, deoxycholic acid; FXR, farnesoid X receptor; LCA, lithocholic acid; MDSC, myeloid-derived suppressor cells; NK T cell, natural killer T cell; NAFLD, non-alcoholic fatty liver disease; PBC, primary biliary cholangitis; PSC, primary sclerosing cholangitis, TNF-α, tumor necrosis factor-alpha; T-reg, T-regulatory cell.

Disruption of the BA-microbiome axis can affect response to ICIs (Routy et al., 2018), summarized in **Figure 2**. Gut microbiota can shape BA composition and activate immune pathways that promote or suppress antitumor immunity (Jia et al., 2018). In turn, BAs can modulate immune cell function—such as T-cell activation or regulatory T-cell expansion. When this network is disrupted (for example, by dysbiosis or altered BA pools), the immune environment may become less favorable for immune checkpoint inhibitors to work effectively (Jia et al., 2018).

Antibiotic treatment can disrupt gut microbiota diversity and composition (gut dysbiosis), potentially diminishing ICI effectiveness in cancer (Routy et al., 2018; Pinato et al., 2019). Broad-spectrum antibiotics can alter key metabolic functions, including the conversion of primary to secondary BAs and the production of immunomodulatory metabolites. Antibiotics can perturb gut bacteria that perform BA biotransformations, shifting primary/secondary bile-acid pools and altering hepatic immune surveillance (for example, via CXCL16–NKT cell signaling), changes that have been directly linked to liver tumor biology in animal models (Wang et al., 2017). Probiotics can help restore microbial functions that influence BA composition, including the conversion of primary to secondary BAs and the regulation of receptors such as FXR and TGR5 that shape hepatic inflammation (Degirolamo et al., 2014). By rebalancing these pathways, certain probiotic strains (such as those enriched with Lactobacillus and Bifidobacterium) may improve gut–liver immune signaling and counter some of the dysbiosis-related changes associated with biliary tract disease (Degirolamo et al., 2014). Beyond BA modulation, probiotics have been shown to suppress tumor-promoting inflammation by reducing proinflammatory cytokines such as IL-6, TNF-α, and IL-17 while enhancing anti-inflammatory mediators including IL-10 and TGF-β, thereby limiting NF-κB–driven oncogenic signaling (O'Callaghan and Sinderen, 2016). In preclinical cancer models, selected probiotic strains also enhance antitumor immunity through improved dendritic cell maturation, increased CD8^+^ T-cell infiltration, and modulation of regulatory T-cell balance, contributing to a tumor microenvironment less permissive to progression (Routy et al., 2018). Additionally, short-chain fatty acid production—particularly butyrate—by probiotic-associated taxa can reinforce epithelial barrier integrity, reduce endotoxemia, and alter myeloid-derived suppressor cell and macrophage polarization, further reshaping the tumor microenvironment toward immune surveillance rather than immune tolerance (Louis and Flint, 2017). Although most mechanistic data derive from colorectal and hepatocellular carcinoma models, these pathways are biologically relevant to BTC given the shared involvement of chronic inflammation, BA dysregulation, and immune escape.

Fecal microbiota transplantation (FMT) from patients with either non-small cell lung cancer or renal cell carcinoma who responded to ICI into germ-free mice has been shown to restore anti–PD-1 efficacy in mice, while FMT from non-responders does not (Routy et al., 2018). This suggests that specific microbial constituents are critical for mediating ICI responsiveness, consistent with clinical observations that taxa such as *Akkermansia muciniphila*, *Ruminococcus callidus*, and *Lachnospiraceae* bacterium-GAM79 are enriched in responders and associated with improved outcomes and longer progression-free survival (Geva-Zatorsky et al., 2017; Routy et al., 2018). **Table 2** below highlights primary studies discussed and their key outcomes.

**Table 2:** Summarization of primary studies cited including sample size and key findings with relevance to BTC.

Study (first author, year)	Study type	Biospecimen(s)	Sample size	Key findings relevant to BTC	Major confounders/limitations
Jia et al. (2020)	Human observational (intrahepatic CCA)	Stool, serum, tumor tissue	~60 ICC; ~30 controls	iCCA is associated with gut dysbiosis, altered bile acid metabolism, and elevated inflammatory cytokines; secondary bile acids correlated with tumor-associated immune patterns.	Cross-sectional design; no causality; potential diet/antibiotic confounding; single-center cohort.
Saab et al. (2021)	Human observational (extrahepatic CCA)	Bile	28 eCCA; 47 benign controls	Biliary dysbiosis in eCCA with enrichment of Enterococcus and Enterobacteriaceae; suggests local microbial contribution to bile acid modification and inflammation.	Small cohort; ERCP sampling contamination risk; controls had biliary disease.
Tsuchiya et al. (2018)	Human observational (gallbladder cancer)	Gallbladder bile	15 GBC; 25 cholelithiasis	Distinct bile microbiota in GBC compared to gallstones; enrichment of taxa implicated in bile acid metabolism.	Small sample size; limited functional/metabolomic validation; regional cohort.
Avilés-Jiménez et al. (2016)	Human observational (extrahepatic CCA)	Bile duct tissue, bile	36 eCCA; benign controls	Increased detection of Helicobacter pylori DNA in CCA tissues; supports microbial involvement in biliary carcinogenesis.	PCR-based detection; viability unclear; association not causation.
Song et al. (2018)	Human metabolomics	Serum	~30 CCA; ~30 controls	Identified glycocholic acid and taurochenodeoxycholic acid as potential CCA biomarkers; indicates altered bile acid profile in malignancy.	Moderate cohort size; lacks mechanistic validation.
Dai et al. (2013)	*In vitro* (CCA cell lines)	Cell culture	Multiple CCA lines	Bile acids promoted CCA cell proliferation via NF-κB activation; inhibition reduced growth.	*In vitro* only; possible supraphysiologic bile acid concentrations.
Erice et al. (2018)	*In vitro* + mouse xenograft	CCA cell lines; murine tumors	Multiple lines; xenograft model	TGR5 activation promoted CCA progression; FXR signaling showed context-dependent modulation of tumor growth.	Preclinical model; pharmacologic agonists may not reflect physiologic exposure.
Wang et al. (2017)	Animal model (cholestasis)	Mouse liver tissue	Bile duct ligation model	S1PR2 mediated bile-acid–induced cholangiocyte proliferation and liver injury; mechanistic link between bile acid signaling and ductular expansion.	Not a cancer model; extrapolation to BTC indirect.
Ma et al. (2018)	Animal model + human correlation	Stool; liver tissue	Murine models; human HCC samples	Microbiota-dependent bile acid metabolism regulated liver cancer via CXCL16–NKT axis; secondary bile acids suppressed antitumor immunity.	Focused on HCC rather than BTC; translational extrapolation required.
Mao et al. (2021)	Human observational (hepatobiliary cancers on anti–PD-1)	Stool	65 patients	Gut microbiome composition associated with response to immunotherapy; supports microbiota–bile acid–immune axis in hepatobiliary cancers.	Mixed hepatobiliary tumors; antibiotic confounding; no direct bile acid measurement.
Zhu et al. (2024)	Human observational (BTC, anti–PD-1/PD-L1)	Stool, serum metabolites	74 BTC patients	Microbial and metabolite patterns predicted immunotherapy response; bile acid–related metabolites associated with outcomes.	Observational; treatment heterogeneity; geographic limitation.

Anti-PD-L1, anti-programmed death ligand 1; BTC, biliary tract cancer; CCA, cholangiocarcinoma; eCCA, extrahepatic cholangiocarcinoma; ERCP, endoscopic retrograde cholangiopancreatography; FXR, farnesoid X receptor; GBC, gallbladder carcinoma; HCC, hepatocellular carcinoma; iCCA, intrahepatic cholangiocarcinoma; NK T cell, natural killer T cell; S1PR2, sphingosine-1-phosphate receptor 2; TGR5, Takeda G protein-coupled receptor 5.

A recent study of 65 patients with advanced hepatobiliary cancers treated with anti–PD-1 therapy found distinct microbial signatures in responders versus non-responders. Responders exhibited enrichment of specific bacterial taxa linked to BA modulation and immune activation (Lee et al., 2022). Similar to the bacterial taxa found in *Routy* et al, in *Mao et al, Lachnospiraceae, Ruminococcus*, and *Erysipelotrichaceae* were associated with enhanced antitumor immunity and increased clinical response to anti–PD-1 treatment for hepatobiliary cancers (Routy et al., 2018; Mao et al., 2021). Of note, *Lachnospiraceae* and *Erysipelotrichaceae* are producers of short-chain fatty acids, which contribute to mucosal immunity by promoting anti-inflammatory cytokine production (e.g., TGF-β, IL-10) and supporting regulatory T cell differentiation (Rooks and Garrett, 2016; Mao et al., 2021). These methods of mucosal immunity have been shown to enhance the effectiveness of PD-1 blockade in colorectal cancer. *Lachnospiraceae* also participates in converting primary to secondary BAs and is considered a beneficial strain for microbiota-based therapies, such as FMT, to restore gut homeostasis and colonization resistance (Mao et al., 2021).

Alpha and beta diversity is also thought to contribute to immunotherapy response in BTC. Alpha diversity describes species richness or diversity within a sample, whereas beta diversity compares the similarity of two or more communities (Cassol et al., 2025). In a prospective cohort study of 88 patients with BTC who received PD-1/PD-L1 inhibitors, alpha diversity in the BTC cohort did not differ significantly between patients with durable clinical benefit and those without at treatment initiation, but beta diversity showed clear separation between groups. This finding aligns with broader evidence that microbial community composition, rather than diversity alone, may be more relevant for predicting ICI efficacy (Fessler et al., 2019; Pinato et al., 2019; Zhu et al., 2024).

Notably, the durable clinical benefit group in the same previously mentioned study by *Zhu et al*, exhibited a higher *Bacteroidetes*/*Firmicutes* ratio, a profile previously associated with favorable immunotherapy responses in other tumor types, such as renal cell carcinoma, non small cell carcinoma, and melanoma (Fessler et al., 2019; Zhu et al., 2024). Within this group, *Bacteroidetes* and particularly *Alistipes* species, including *A. putredinis* and *A. dispar*, were enriched. High *Alistipes* abundance correlated with longer overall and progression-free survival, consistent with observations in other malignancies (Jin et al., 2019; Zhu et al., 2024). Mechanistically, *Alistipes* may enhance antitumor immunity by producing short-chain fatty acids, modulating inflammatory pathways, and influencing immune cell recruitment. **Table 3** summarizes clinical cohorts discussed and their associations with microbial taxa and metabolite profiles.

**Table 3:** Summary of clinical cohorts and preclinical models evaluating associations between gut microbial taxa, metabolite profiles, and response to PD-1/PD-L1–based immunotherapy in biliary tract and other solid tumors.

Study/cohort	Cancer type	ICI regimen	sample size	Response definition	Key taxa/metabolites associated with response	Notes
Routy et al. (FMT study)	NSCLC; RCC	Anti–PD-1	153 patients	Clinical response to anti–PD-1; responder vs non-responder FMT donors	*Akkermansia muciniphila*, *Ruminococcus callidus*, Lachnospiraceae bacterium-GAM79	FMT from responders restored anti–PD-1 efficacy in germ-free mice; non-responder FMT did not (preclinical validation of clinical association).
Mao et al.	Advanced hepatobiliary cancers	Anti–PD-1	65 patients	Responder vs non-responder (anti–PD-1)	Lachnospiraceae, Ruminococcus, Erysipelotrichaceae	Enriched taxa linked to BA modulation and immune activation; overlaps with Routy taxa.
Zhu et al. (prospective cohort)	Biliary tract cancer	PD-1/PD-L1 inhibitors	88 patients	DCB vs non-DCB	Higher Bacteroidetes/Firmicutes ratio; enrichment of Alistipes spp. (*A. putredinis*, *A. dispar*)	Alpha diversity is not different; beta diversity separates DCB vs non-DCB. High *Alistipes* associated with longer OS and PFS.

anti-PD-L1, anti-programmed death ligand 1; BA, bile acid; DCB, durable clinical benefit; FMT, fecal microbiota transplantation; NSCLC, non-small cell lung cancer; OS, overall survival; PFS, progression free survival; RCC, renal cell carcinoma; SCFA, small chain fatty acids.

These results suggest that specific microbiome signatures, especially enrichment of *Alistipes* and a higher *Bacteroidetes*/*Firmicutes* ratio, may predict and potentially augment ICI responsiveness in BTC. Modulation of gut microbiota through interventions such as probiotics or fecal microbiota transplantation could represent a future strategy to improve immunotherapy efficacy.

Recent studies have revealed a novel class of microbially conjugated BAs, in which gut bacteria link canonical amino acids such as phenylalanine, tyrosine, or leucine to BAs (Garcia et al., 2022). These microbially conjugated BAs may alter host–microbiome communication, immune signaling, and BA receptor interactions in ways not yet fully understood (Garcia et al., 2022).

## Common confounding clinical factors

Interpretation of BA–microbiota associations in BTC must account for clinical exposures that can substantially reshape both bile acid composition and microbial community structure. Biliary obstruction and cholestasis, common in both intrahepatic and extrahepatic disease, alter intraductal BA concentrations, increase hydrophobic BA retention, and disrupt the protective “bicarbonate umbrella,” thereby promoting epithelial injury and inflammation independent of tumor biology (Beuers et al., 2010; Chiang, 2013). Endoscopic or percutaneous biliary drainage further modifies local oxygen tension and introduces procedural contamination, potentially shifting biliary microbial profiles. Episodes of cholangitis can acutely enrich Proteobacteria and other pathobionts, complicating interpretation of dysbiosis as causative versus reactive. Proton pump inhibitors and metformin independently reshape gut microbial composition and BA pools, while ursodeoxycholic acid directly modifies the hydrophobicity index and receptor signaling milieu of the BA pool (Fiorucci et al., 2009; Chiang, 2013). Diet, inpatient status, and recent hospitalization introduce additional variability, influencing microbial diversity metrics and BA metabolism (Rooks and Garrett, 2016; Cassol et al., 2025). Future studies should therefore incorporate rigorous reporting of biliary instrumentation, recent cholangitis, antibiotic and PPI exposure, antihyperglycemic therapy, ursodeoxycholic acid use, oncologic treatment, dietary patterns, and hospitalization history. Prospective sampling prior to biliary intervention when feasible, stratification by obstruction status, standardized timing relative to antibiotic exposure, concurrent BA metabolomics, and transparent reporting of alpha/beta diversity methodology are essential to disentangle tumor-associated signals from treatment-related perturbations. Such design considerations will improve reproducibility and allow clearer attribution of microbial and BA alterations to BTC pathobiology rather than clinical confounders.

## Future directions and conclusion

The interplay between the gut microbiota and BAs represents a dynamic, bidirectional system with far-reaching implications for the pathogenesis, diagnosis, and treatment responsiveness of BTCs. Microbial transformations of BAs alter their physicochemical properties and signaling potential, with downstream effects on antimicrobial activity, epithelial barrier function, inflammation, and immune surveillance. However, much of the current evidence derives from cross-sectional or heterogeneous hepatobiliary cohorts, underscoring the need for more rigorous and mechanistically anchored study designs.

As the evidence grows linking BA dysregulation to cancer progression and therapy resistance, new therapeutic opportunities are emerging. Future research should prioritize longitudinal sampling before and after biliary interventions (e.g., stenting, drainage, antibiotics, chemotherapy) to disentangle tumor-associated signals from procedure- or obstruction-related effects. Paired bile and stool sampling with integrated multi-omics (metagenomics, targeted and untargeted metabolomics, transcriptomics) will be essential to define compartment-specific microbial and BA profiles and their convergence on receptor signaling pathways. Targeted BA panels stratified by conjugation status and primary versus secondary species should be linked to functional readouts of FXR, TGR5, S1PR2, and related receptor activity in cholangiocytes, hepatocytes, and immune cells. Mechanistic validation using patient-derived organoids, primary cholangiocyte models, and gnotobiotic or humanized mouse systems colonized with defined microbial consortia will be critical to establish causality rather than association.

Although microbiota-directed therapies (probiotics, selective antibiotics, fecal microbiota transplantation) and BA-targeted agents (e.g., FXR agonists) are conceptually attractive, their translation into BTC requires careful evaluation of feasibility and risk. In patients with biliary obstruction, cholangitis, or immunosuppression, microbial manipulation may increase the risk of bacteremia or sepsis. BA receptor agonists may exert context- and cell type–specific effects that differ across BA species and tumor subtypes. Early-phase trials should therefore incorporate safety endpoints, standardized definitions of biliary infectious complications, and mechanistically informed biomarkers (e.g., receptor activation signatures, BA composition shifts, immune profiling). Clinically meaningful endpoints to justify further translation would include improvements in progression-free survival, enhancement of immunotherapy response rates, reduction in cholangitis frequency, or reproducible modulation of validated BA–receptor signaling pathways.

A coordinated strategy integrating longitudinal human cohorts, compartment-resolved multi-omics, and functional modeling will be necessary to determine whether modulation of the microbiota–BA axis can move from biological plausibility to clinically actionable therapy in BTC.
